# Dirac-vortex topological photonic crystal fibre

**DOI:** 10.1038/s41377-020-00432-2

**Published:** 2020-12-22

**Authors:** Hao Lin, Ling Lu

**Affiliations:** 1 Institute of Physics, Chinese Academy of Sciences/Beijing National Laboratory for Condensed Matter Physics, Beijing, 100190 China; 2 School of Physical Sciences, University of Chinese Academy of Sciences, Beijing, 100049 China; 3 Songshan Lake Materials Laboratory, Dongguan, 523808 Guangdong, China

**Keywords:** Photonic crystals, Micro-optics

## Abstract

The success of photonic crystal fibres relies largely on the endless variety of two-dimensional photonic crystals in the cross-section. Here, we propose a topological bandgap fibre whose bandgaps along in-plane directions are opened by generalised Kekulé modulation of a Dirac lattice with a vortex phase. Then, the existence of mid-gap defect modes is guaranteed to guide light at the core of this Dirac-vortex fibre, where the number of guiding modes equals the winding number of the spatial vortex. The single-vortex design provides a single-polarisation single-mode for a bandwidth as large as one octave.

## Photonic crystals: Designing novel optical fibers with topological control

Numerical calculations reveal how optical fibers composed of topological photonic crystals could allow improved versatility and control over the modes and polarizations of the light they transmit. Photonic crystals have compositions that create “bandgaps” preventing the passage of light with waves of specific energies and momenta. Hao Lin and Ling Lu at the Institute of Physics of the Chinese Academy of Sciences, base their novel method to transmit pure “single mode” light, over a large frequency range, using a topological feature known as a “Dirac-vortex”. In principle, it could lead to applications that can transmit light signals more stably over long distances. At present, the work is theoretical, but the authors suggest that the fibers could be made from silica using existing stack-and-draw or 3-D printing technologies. Fabrication and testing are the next desirable steps.

## Introduction

Topological photonics^1–3^, initiated with the idea of robust waveguiding, is inspiring novel fibre concepts, such as a one-way fibre inside a magnetic three-dimensional photonic crystal^4^ and a Bragg fibre with nontrivial edge modes^5^. In this article, we introduce the topological photonic crystal fibre (PCF) whose invariant cross-section resembles the recent Dirac-vortex topological cavity^6^ in two-dimensional photonic crystals. Such a topological bound state can be traced back to the Jackiw-Rossi zero mode in the two-dimensional (2D) Dirac equation^7^ and has been realised in honeycomb lattices^8,9^ in a couple of systems^10–13^. This Dirac-vortex silica fibre can support an *arbitrary* number of nearly degenerate guiding modes by varying the winding number (*w*) of the spatial vortex. When *w* = ±1, the fibre can support a single-polarisation single-mode (SPSM) with a large bandwidth.

The SPSM fibre supports truly one mode, while traditional single-mode fibres and polarisation-maintaining fibres both support two polarisations, either degenerate or nondegenerate, respectively. Such fibre birefringence (dual polarisation) broadens the optical pulses being transmitted, known as polarisation-mode dispersion. To solve this limitation, SPSM fibres have been designed to separate the degenerate cutoff frequencies by lowering the symmetry of the fibre cross-section, which can be achieved by structural asymmetry or nonuniform stress. This dominant asymmetric approach is applied mostly to the lowest-frequency index-guided fundamental modes (polarisation-degenerate)^14–19^ but also works for the nonfundamental degenerate modes inside a bandgap^20–22^.

**Fig. 1:**
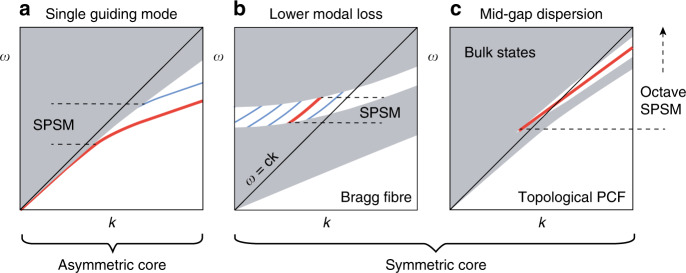
Asymmetric and symmetric approaches to an SPSM. **a** Dominant approach to split the degenerate fundamental modes by structural asymmetry. **b** Proposed Bragg fibre design to guide light by a singly degenerate mode with lower loss than other modes. **c** The topological PCF provides singlet mid-gap dispersion for a broadband SPSM

The SPSM bandwidth is very limited due to the amount of asymmetry that can be applied; the best experimental value is ~30% (frequency span over central frequency), which is achieved in a stressed PCF^18^ (In integrated waveguides, an SPSM bandwidth over one octave [66.67%] has been demonstrated^23^). For example, in Fig. 1a, the SPSM bandwidth is bounded from above by the cutoff frequency of the other polarisation (blue) and bounded from below by the confinement loss, even if this guiding mode (red line) has no cutoff frequency. However, there exists a symmetric approach to an SPSM by operating at a singly degenerate mode inside a bandgap so that the fibre cross-section can remain highly symmetric. Shown in Fig. 1b, this symmetric approach has thus far only been proposed theoretically in a hollow-core Bragg fibre^24,25^, in which many other guided modes (blue line) can be made much lossier than the TE_01_ mode (red line). The SPSM bandwidth of this design is even more limited.

The Dirac-vortex fibre is an ideal design for an ultrabroadband SPSM by ensuring singlet mid-gap dispersion inside the bandgap (the symmetric approach), as illustrated in Fig. 1c. Without the topological mechanism of the Dirac-vortex, it is generally difficult to stabilise a defect mode at the middle of the bandgap for every wave vector.

In the rest of the article, we first show how to gap the nodal-line without leaving residual Weyl points. Such a supercell Kekulé modulation^6,8^ is generalised to continuous 2*π* phase angles that are used to construct a vortex gap around the fibre core that can confine any number of fibre modes. To ease the fabrication, a simplified design of only four capillary silica tubes is introduced. Finally, we enlarge the vortex size to eliminate the index-guided modes and achieve an octave-spanning SPSM.

## Results

### Nodal lines and Weyl points in a PCF

**Fig. 2:**
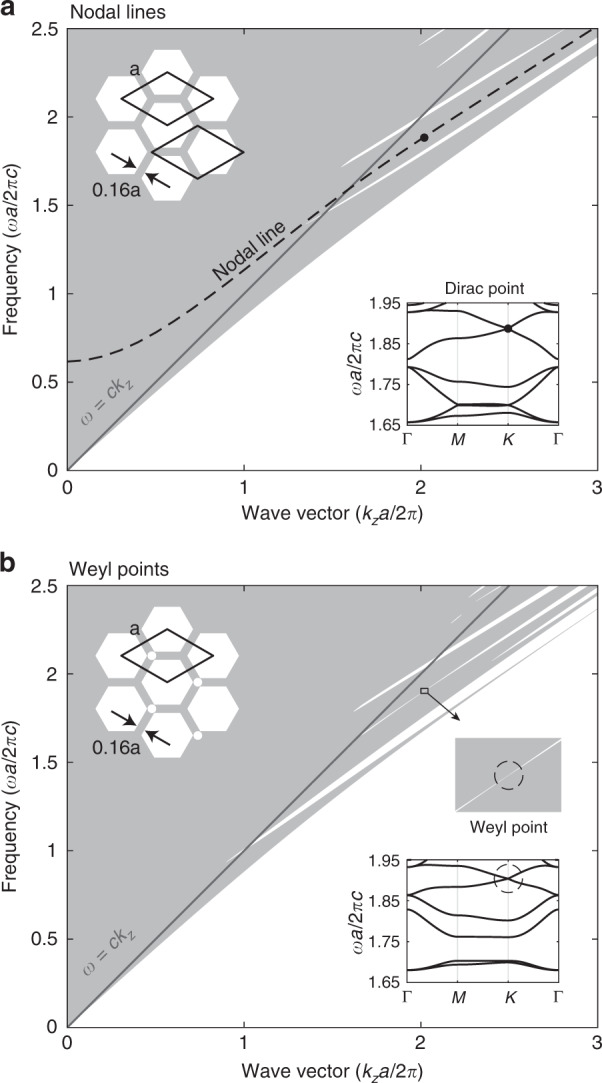
Band diagram of silica (*ε* = 2.1) photonic crystal lattices uniform in the out-of-plane direction (*z*). **a** Projected band diagram of the triangular photonic crystal, in which the nodal-line degeneracy is highlighted. **b** An extra air hole in the primitive-cell breaks the inversion symmetry, and the nodal-line is lifted into Weyl points. Insets: cross-section structures and in-plane band structures at *k*_*z*_*a*/2*π* = 2.02. Two different primitive-cell choices are drawn in **a**

We start with the most common PCF structure^26,27^, a silica photonic crystal with a triangular lattice of air holes, shown in Fig. 2a. There are two nodal lines of 2D Dirac points^28,29^ at the ±*K* points in the Brillouin zone. The Dirac points are frequency isolated in the in-plane 2D band structure for *k*_*z*_*a*/2*π* > 1.6. Each nodal-line gaps into Weyl points^30^ if we break the inversion symmetry by adding an extra small air hole in the primitive cell, as shown in Fig. 2b. Since this PCF structure has mirror symmetry along *z*, the Weyl surface state does not have handedness propagating along the *z* axis, unlike that observed in coupled spiral waveguide arrays^31,32^ containing similar type-II Weyl points. To gap the entire nodal-line, without leaving Weyl degeneracies, we apply supercell modulations.

### Generalised Kekulé modulation

**Fig. 3:**
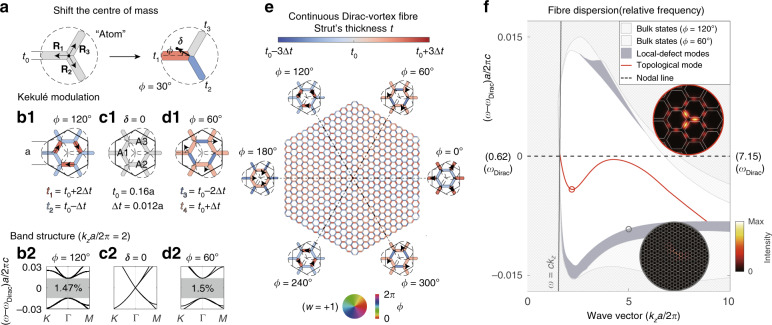
Dirac-vortex fibre obtained by continuous Kekulé modulations. **a** Example of how an “atom” can be shifted in any direction (arg[***δ***] = *ϕ*) with finite amplitude (|***δ***|) by changing the widths of the three struts. (**b1**), (**c1**), (**d1**) Supercell examples of three coordinated atoms (A1, A2, and A3) with $|{\boldsymbol{\delta}}| = {\sqrt 3} a/80$, *ϕ* = 120°; |***δ***| = 0; and $|{\boldsymbol{\delta}}| = {\sqrt 3} a/80$, *ϕ* = 60°, respectively. $\Delta {t} = 2{\sqrt 3} |{\boldsymbol{\delta}}|t_{0}/a = 0.012a$. The corresponding band structures are plotted in (**b2**), (**c2**), and (**d2**), respectively. **e** Structure of a continuous Dirac-vortex PCF, in which every strut is coloured according to its width. **f** Band diagram of the fibre plotted in reference to the frequency of the original nodal-line (central dashed line). The inset shows the intensity patterns ($\widehat {\boldsymbol{z}} \cdot {\mathrm{Re}}[{\boldsymbol{E}}^ \ast \times {\boldsymbol{H}}]$) of the topological mode and one local-defect mode. The single-polarisation topological mode (red line) spans over two octaves

As shown in Fig. 3a–d, we perturb the nodal-line lattice with the generalised Kekulé pattern discussed in ref. ^6^. The idea is to couple the two nodal lines (of Dirac points) together in an enlarged supercell and annihilate them into a bandgap through supercell modulation. Since each supercell has three primitive cells, we label each primitive cell [Fig. 2a inset] as an artificial “atom” consisting of three struts [Fig. 3a]. In practice, we shift the three “atoms” in the supercell with identical amplitude and constant phase difference (120°) between each two “atoms”. To move each “atom”, we can shift its centre of mass ***δ***(*ϕ*), in any direction *ϕ*, by adjusting the thickness of the three struts (*t*_1_, *t*_2_, *t*_3_) without changing the total mass of the “atom” (∝3*t*_0_). Therefore, the strut thickness for any modulation vector ***δ***(*ϕ*) can be determined by the following equation:

1 $$\begin{array}{*{20}{l}} {\sum \limits_{i = 1}^3} {t_i} {\mathbf{R}}_i = {\boldsymbol{\delta}} \left( \phi \right){\sum \limits_{i = 1}^3} {t_i} = {\boldsymbol{\delta}} \left( \phi \right)3t_0 \end{array}$$

where **R**_*i*_ ($|{\mathbf{R}}_i| = \sqrt 3 /6a$) is the position vector of the centre of mass of each strut. Examples of the lattices before and after the Kekulé modulations are drawn in Fig. 3b1–d1, and their corresponding band structures are plotted in (b2–d2).

After the generalised modulation (|***δ***(*ϕ*)| ≠ 0), the entire nodal lines gap out for arbitrary *ϕ* ∈ (0, 2*π*), and the bandgaps share a common frequency range, which is eventually determined by the band edges of the two most symmetric supercells of *ϕ* = 60° and 120°. The lattices of the rest of the modulation phases can be understood as interpolations between the two. Note that the modulation phase *ϕ* of each supercell is labelled by the shift angle of “atom” A1 in Fig. 3c1.

### Continuous modulation

As shown in Fig. 3e, we arrange the series of Kekulé-modulated 2D lattices angularly around a chosen core point in the fibre cross-section as a function of their modulation phase *ϕ*. As a result, a single defect mode appears in the middle of the bandgap and is spatially localised at the fibre core, as shown in Fig. 3f. This result is expected from the Dirac-vortex cavity result^6^ because a PCF can be regarded as a 2D cavity for every propagation constant (*k*_*z*_). Since the fibre is *C*_3*v*_ symmetric, one-sixth of the structure is sufficient to study this topological mode.

The large momentum (*k*_*z*_*a*/2*π* > 5), or short-wavelength, behaviour of the fibre band diagram in Fig. 3f is worth discussion. First, the common bandgap frequency is lower than that of the nodal-line (central dashed line in the figure) at short wavelengths because the short-wavelength modes can localise preferentially at struts that are thicker than *t*_0_ (rather than at those that are thinner) in the modulated lattice, resulting in lowering of the overall band frequencies. Second, the topological mode is very sensitive to the central three struts, where the modal intensity peaks. In this case, the three central red struts are the thickest, and consequently, the fibre mode cuts off, in the short-wavelength region, at a frequency lower than the bandgap. Third, although we “continuously” vary the modulation angle *ϕ* in the surrounding lattice, the discretization is still limited to a single strut in our design. A consequence of the strut-discrete vortex is that a set of extra modes is trapped very close to the spectral edges of the bandgap. We call them local-defect modes, shaded in grey colour in the figure. These modes tend to arise at short wavelengths and are more prominent for large modulation amplitudes (large bandgaps), as shown in the example in Fig. 3f. Since these local-defect modes are located near the bulk band edges with an extended mode profile, their confinement losses are at least one order of magnitude higher than that of the guided topological mode with *w* = +1.

### Arbitrary degenerate modes

**Fig. 4:**
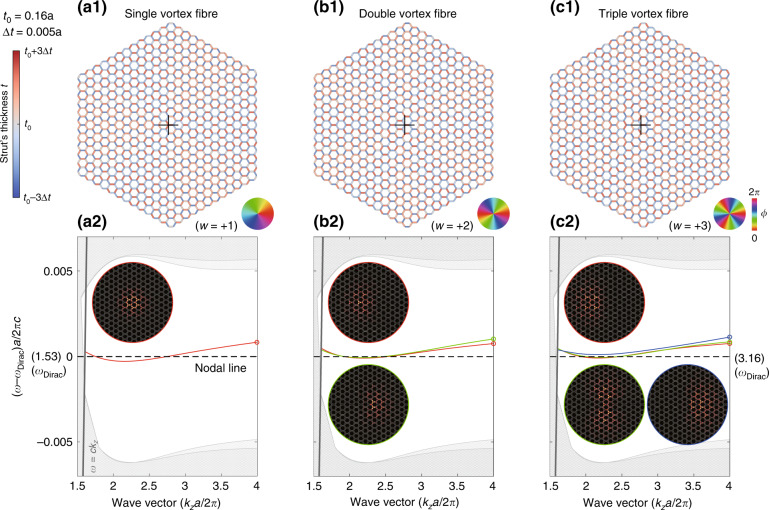
Continuous Dirac-vortex fibres with winding number *w* = +1, +2, +3. (**a1**), (**b1**), and (**c1**) are the fibre structures. The colour wheels show the corresponding phases of the generalised Kekulé modulation. (**a2**), (**b2**), and (**c2**) are the corresponding band diagrams, and the insets show the mode profiles ($\widehat {\boldsymbol{z}} \cdot {\mathrm{Re}}[{\boldsymbol{E}}^ \ast \times {\boldsymbol{H}}]$) of modes at *k*_*z*_*a*/2*π* = 4

One main topological feature of the Dirac-vortex fibre is the ease of creating multiple near-degenerate modes by simply increasing the winding number (*w*) of the vortex^6^. We demonstrate this in Fig. 4 for *w* = +1, +2, +3, where *w* is the topological invariant of the system and can be an arbitrary integer. The sign of *w* determines where the field peaks around one of the two sublattices of a honeycomb lattice. The two sublattices can be viewed as the two joints, each having three struts, in the primitive cell of Fig. 2a. An example of *w* = −1 is presented in Supplementary Part I.

In these multimode examples, we decrease the modulation amplitude (as well as the bandgap size) to eliminate the local-defect modes in the momentum range plotted. Additionally, we keep the core centre at the *C*_3*v*_ centre of the *w* = +1 vortex. If we choose a *w*-dependent centre, then any vortex can be *C*_3*v*_ symmetric, so some pairs of fibre dispersions will be rigorously degenerate due to the doublet representation of the point group.

In principle, a continuous, single-mode or multimode, Dirac-vortex PCF can be fabricated either from three-dimensional (3D)-printed preforms or by the stack-and-draw method with over a hundred tubes with different tube thicknesses. Neither of the two solutions are convenient. Therefore, we present the discrete version of the fibre design.

### Discrete modulation with four tubes

We discretize the continuous vortex design [Fig. 3e] into six discrete modulation phase angles (*ϕ*). Owing to the *C*_3*v*_ symmetry of the cross-section, only two phases of the six have distinct structures. The three phases that differ by 120° are actually identical in the unit-cell structure (but with different origins and orientations). As a result, we only need four tubes to stack-and-draw the Dirac-vortex PCF, which is very reasonable for fabrication. Another discrete version is presented in Supplementary Part II using four strut thicknesses.

**Fig. 5:**
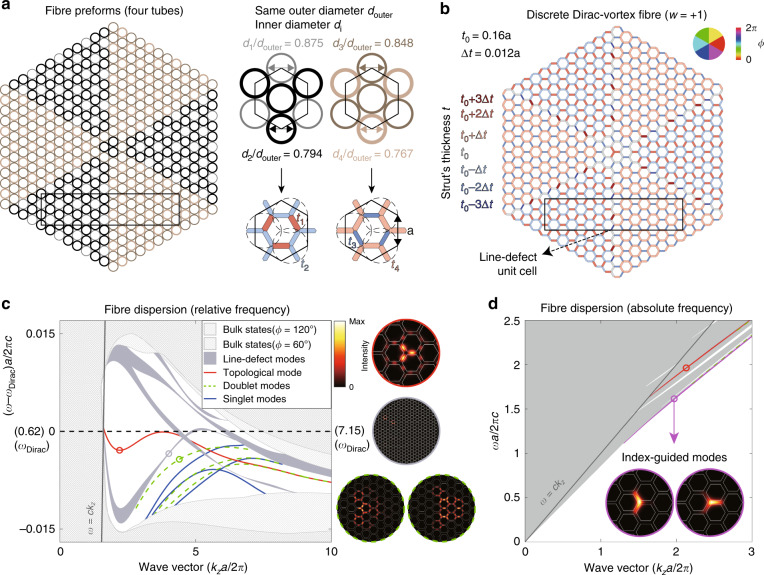
Dirac-vortex fibre obtained by discrete Kekulé modulations. **a** Fibre preform with four capillary silica tubes of the same outer diameter. **b** Fibre structure, having seven different strut widths, resulting from the preform in **a**. The black rectangle is the unit cell for the line-defect modes. **c** Band diagram with the frequency referenced to the original nodal-line of the unmodulated lattice. The higher-order modes are plotted with blue and green lines according their degeneracy. Insets show the mode profiles ($\widehat {\boldsymbol{z}} \cdot {\mathrm{Re}}[{\boldsymbol{E}}^ \ast \times {\boldsymbol{H}}]$). **d** Purple dispersion and mode profiles of the polarisation-degenerate index-guided fundamental mode. Other index-guide modes are plotted with green dashes

As shown in Fig. 5a, the four silica capillary tubes have the same outer diameter *d*_outer_ to maintain the lattice but different inner diameters *d*_*i*_ for modulation. The four exact ratios of *d*_*i*_/*d*_outer_ can be determined from Eq. 2, satisfying the thickness and area proportionality.

2 $$\left\{ {\begin{array}{*{20}{l}} {\displaystyle \frac{{d_{{\mathrm{outer}}} - (d_{1,4} + d_{2,3})/2}}{{t_{2,4}}}}={\frac{{d_{{\mathrm{outer}}} - d_{2,3}}}{{t_{1,3}}}} \hfill \\ {\displaystyle \frac{{\pi \left( {3d_{{\mathrm{outer}}}^2 - d_{1,4}^2 - 2d_{2,3}^2} \right)}}{{4d_{{\mathrm{outer}}}^2}}}={\frac{{3\sqrt 3 t_0\left( {a - t_0/2} \right)}}{{a^2}}} \hfill \end{array}} \right.$$

The resulting discrete Dirac-vortex fibre and its band structure are plotted in Fig. 5b, c. Compared to the continuous version in Fig. 3e, f, the structural nonuniformity now only exists at the six identical interfaces (also determined by Eq. 2) between the two distinct lattices^33^, at which the extra defect modes can locate. In addition to these line-defect modes inside the bandgap, there are also higher-order vortex modes plotted in green and blue lines, according to their *C*_3*v*_ point group representations. The mode profiles of the higher-order modes are much larger than that of the topological mode.

We have been focusing on the modes inside the bandgap. However, as shown in Fig. 5d [also in Fig. 3e, f], there could also be index-guided modes in the Dirac-vortex fibre whose frequency is the lowest for a common wave vector. Index-guided modes exist wherever there is a sharp local maximum of the strut thickness, equivalent to a local rise of the effective refractive index. The loss of the index-guided modes is usually much lower than that of the in-gap modes.

### Nonzero vortex size with continuous modulation

To operate at the topological mid-gap dispersion, we have to remove the index-guided modes in the design. We do this by enlarging the vortex size and smoothing the sharp lattice (strut) interfaces at the fibre core. The smooth modulation envelope function is Δ*t*(*r*) = tanh(*r*/*R*), where *r* is the radial distance and *R* is the vortex radius of choice, similar to the 2D cavity design in ref. ^6^. Note that in the previous examples of this paper, the vortex size is *R* = 0.

**Fig. 6:**
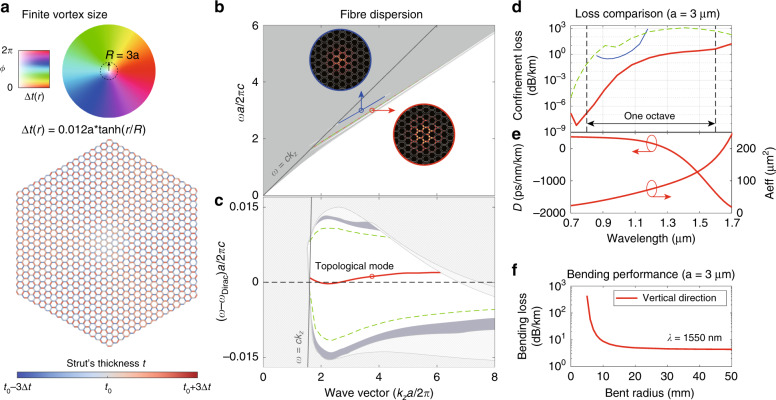
Octave SPSM in a continuous Dirac-vortex fibre with a finite vortex size. **a** Fibre structure with 16 cladding periods in radius. The colour wheel represents the phase and amplitude of the generalised Kekulé modulation. **b** Complete fibre dispersion in absolute frequency. First topological mode (red line) and higher-order doublet modes (green dotted line) in the first topological bandgap, as well as a second topological mode (blue line) in the second topological bandgap at higher frequency. The mode profiles ($\widehat {\boldsymbol{z}} \cdot {\mathrm{Re}}[{\boldsymbol{E}}^ \ast \times {\boldsymbol{H}}]$) of the two topological modes are shown in the insets, circled with different colours for clarity. **c** Fibre dispersion in frequency relative to the original nodal-line frequency. **d** Confinement losses of the guided modes. **e** Dispersion parameter and effective area of the first topological mode. **f** Bending loss of the first topological mode at *λ* = 1550 nm

The fibre cross-section with vortex size *R* = 3*a* is shown in Fig. 6a, and the corresponding band structure is shown in Fig. 6b, c. There are no index-guided modes at the bottom of the bands. For completeness, we also plot the blue mid-gap dispersion inside the second topological bandgap, originating from a higher frequency Dirac point (nodal-line) illustrated in Supplementary Part III. We did not discuss it in the previous examples because it has a much higher loss than the red mid-gap modes in the first topological gap, as shown in Fig. 6d.

### Octave SPSM

The continuously modulated Dirac-vortex PCF with a finite vortex diameter (2*R* = 6*a*) has an SPSM. We evaluate its potential performance in terms of the confinement loss, dispersion parameter, effective area and bending loss in Fig. 6d–f. We choose the wave vector (*k*_*z*_*a*/2*π*) range from 1.8 to 5.7 and the period *a* = 3 μm so that the corresponding wavelength ranges from 1700 nm to 700 nm—the low-loss window of silica.

The modes with the lowest confinement losses are plotted in Fig. 6d, computed with absorbing boundary conditions. The loss of the topological mode (red) is always the lowest for the whole wavelength range over one octave. Most of its confinement loss is less than 1dB/*km* with sixteen layers of air holes along the fibre radius [Fig. 6a].

The dispersion parameter *D* of the topological mode can take large negative values of −1500 ps/nm/km, and the zero-dispersion wavelength is ~1.3 μm. The effective area (*A*_eff_ defined in ref. ^34^) is 23 μm^2^ at a 0.7 μm wavelength and increases to 243 μm^2^ at a 1.7 μm wavelength. The bending loss is calculated using a conformal transformation analysis^35^ with an equivalent refractive index profile $n_{eq}(x,y) = n(x,y)\exp (x/X)$^36^ for bending radius *X*. The results in Fig. 6f show that the fibre can be bent to a radius as small as 15 mm without experiencing significant losses. These specifications of the Dirac-vortex PCF are similar to those of previous PCFs^37,38^, with the key difference being that the topological mode is a single-polarisation mode.

## Discussion

We numerically investigate the Dirac-vortex topological PCF in terms of its principle, construction, and potential performance. This fibre could be made with the standard stack-and-draw process with silica glass tubes or 3D-printed preforms. The design is tolerant of structural details such as the interface curvatures, not considered in the main text but discussed in Supplementary Part IV. It is also possible to move the topological dispersion above the light line, as discussed in Supplementary Part V.

Similar to the case of a three-dimensional one-way fibre^4^, where the topological invariant is a four-dimensional second Chern number, the topological invariant of the Dirac-vortex PCF can be written as a three-dimensional integral (equation 4.2 in ref. ^39^ under chiral symmetry) where the coordinates are *k*_*x*_, *k*_*y*_ and the polar angle (a form of synthetic dimension^40^).

The advantage of the Dirac-vortex PCF over previous fibres is the ability to guide any number of near-degenerate modes at will. The single-mode design provides an SPSM fibre with an octave bandwidth. The effective mode area is easily tuneable by changing the vortex size (*R*). This work suggests PCFs as a new platform for topological photonics.

We are aware of a relevant work after paper submission^41^.

## Supplementary information

Supplementary
