# Supplementary material for: Dirac-vortex topological photonic crystal fibre
Source: Light Sci Appl. 2020 Dec 22;9:202. doi: 10.1038/s41377-020-00432-2 (PMC7755907; doi:10.1038/s41377-020-00432-2)
Supplement: Supplementary file 1 — Supplementary [file 41377_2020_432_MOESM1_ESM.pdf]

Supplementary information for "Dirac-vortex topological photonic crystal fibre"

Hao Lin[1,2] and Ling Lu[1,3]

[1]*Institute of Physics, Chinese Academy of Sciences/Beijing National Laboratory for Condensed Matter Physics, Beijing 100190*

[2]*School of Physical Sciences, University of Chinese Academy of Sciences, Beijing 100049, China*

[3]*Songshan Lake Materials Laboratory, Dongguan, Guangdong 523808, China*

Abstract

CONTENTS

I. The sign of winding number 2

II. Discrete modulation using four strut thicknesses 3

III. High-frequency Dirac point 4

IV. Design tolerance 5

V. Operation above light line 7

References 8

I. THE SIGN OF WINDING NUMBER

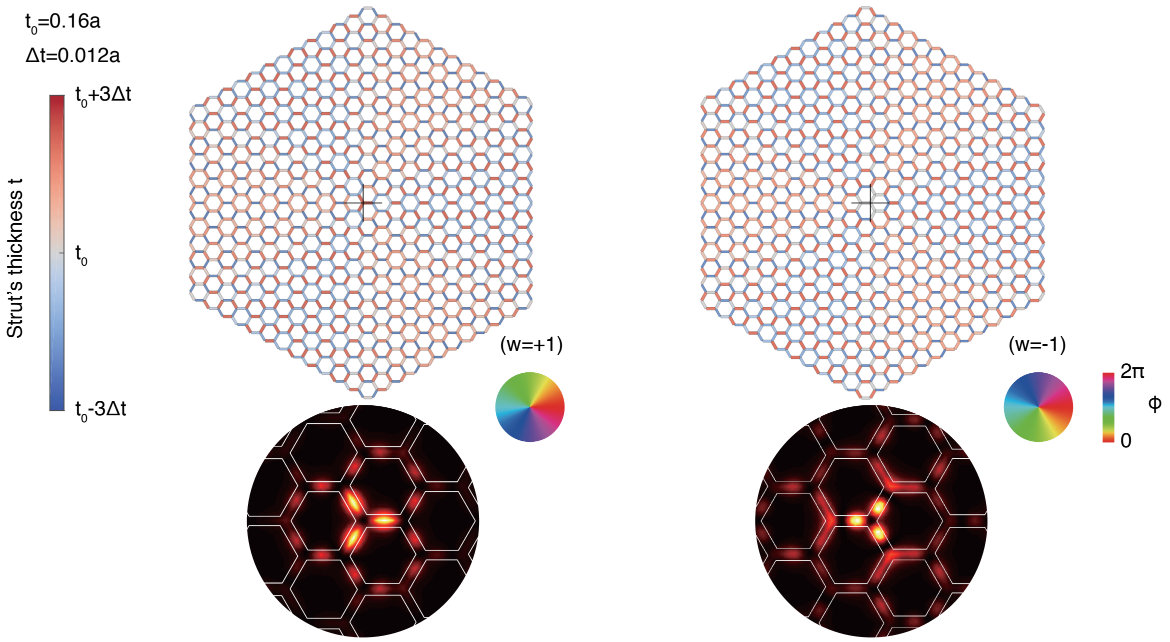

FIG. S1. Comparison of the field distributions for opposite winding numbers of the Dirac-vortex PCF. The intensity patterns localize on different strut joints, which are the two sub-lattices in a honeycomb lattice.

II. DISCRETE MODULATION USING FOUR STRUT THICKNESSES

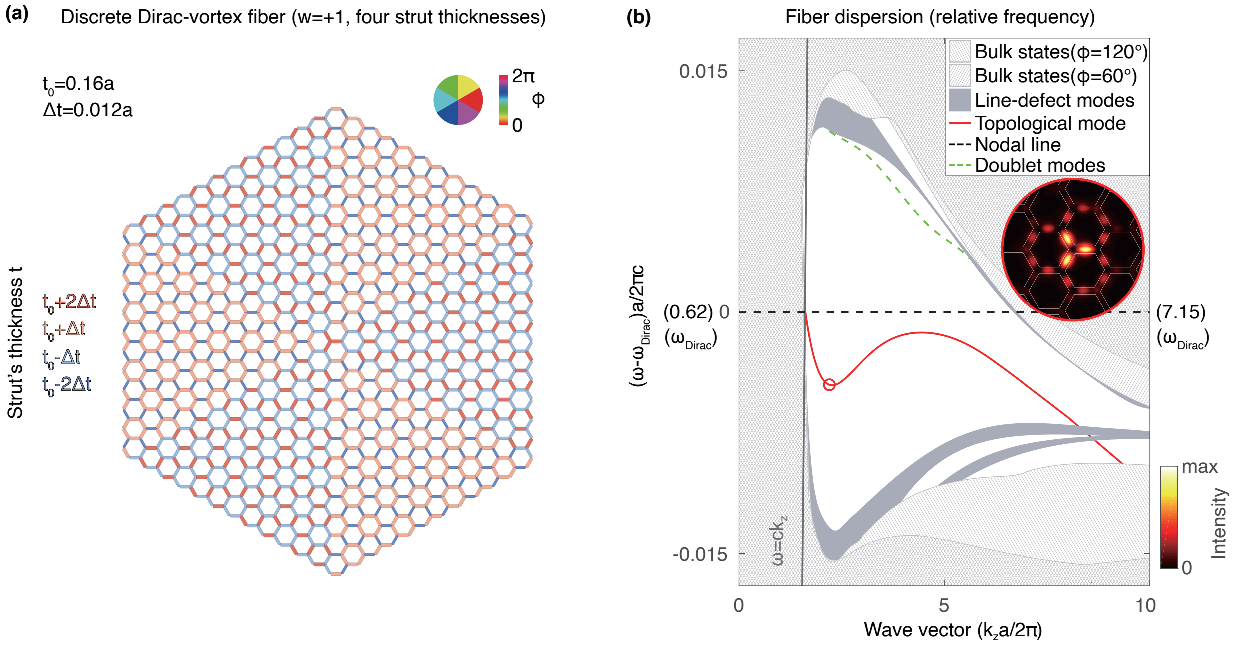

FIG. S2. Discrete Dirac-vortex fiber constructed by four strut thicknesses, instead of four tubes in Fig. 5 in the main-text. The cleaner band diagram is due to the lack of extra strut thicknesses from the tube construction of the stack-and-draw technique.

III. HIGH-FREQUENCY DIRAC POINT

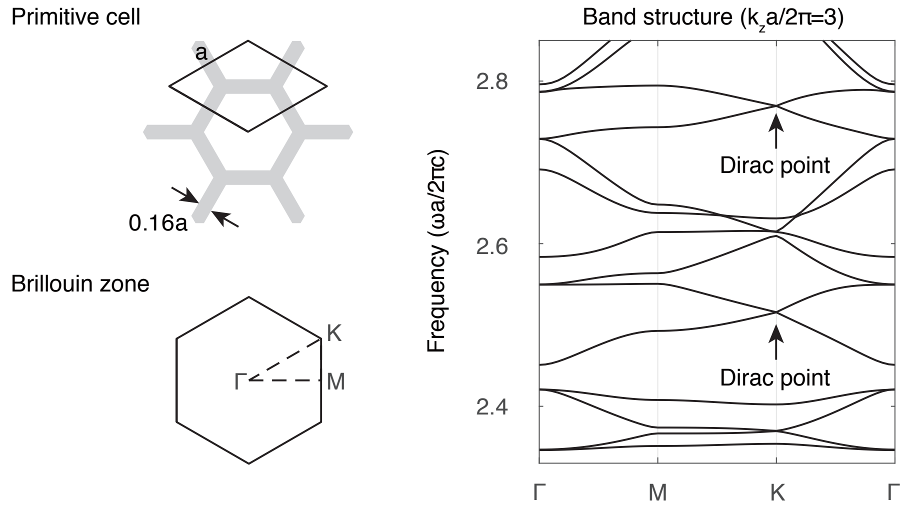

FIG. S3. Band structure of the honeycomb primitive cell at $k_z a/2\pi = 3$. The two frequency-isolated Dirac points correspond to the two topological dispersions in Fig. 6(b) in main text.

IV. DESIGN TOLERANCE

Unlike the other topological waveguides, the Dirac-vortex fiber does not support one-way propagation nor sharp corner turning. The stability of the Dirac-vortex PCF lies in the design itself. First of all, this is essentially a coreless fiber [1] where the defect is not created by adding nor removing materials locally. The topological defect of the vortex is formed by gently perturbing the whole lattice globally, so that small local fabrication imperfection cannot unwind the vortex nor the vortex mode. Secondly, it is well known that the fiber drawing process will smoothen all the sharp edges, which we have not considered for the $120°$ corners between the neighboring two struts in the design. If we allow the rounded corners in the modeling [2, 3], the thickness difference between the struts decreases, so does the modulation amplitude responsible for the gap opening. Consequently, although the topological gap shrinks as the curvature radius increases, it remains open for typical curvature values, as show in Fig. S4(a). Structure in Fig. S4(b) is a discrete vortex fiber (from four-tube construction) with curvature radius $r' = 0.3a$, obtained by adding extra material to the design in Fig. 5(b) in the main-text. The corresponding dispersion and loss performance are shown in Fig. S4(c) and Fig. S4(d).

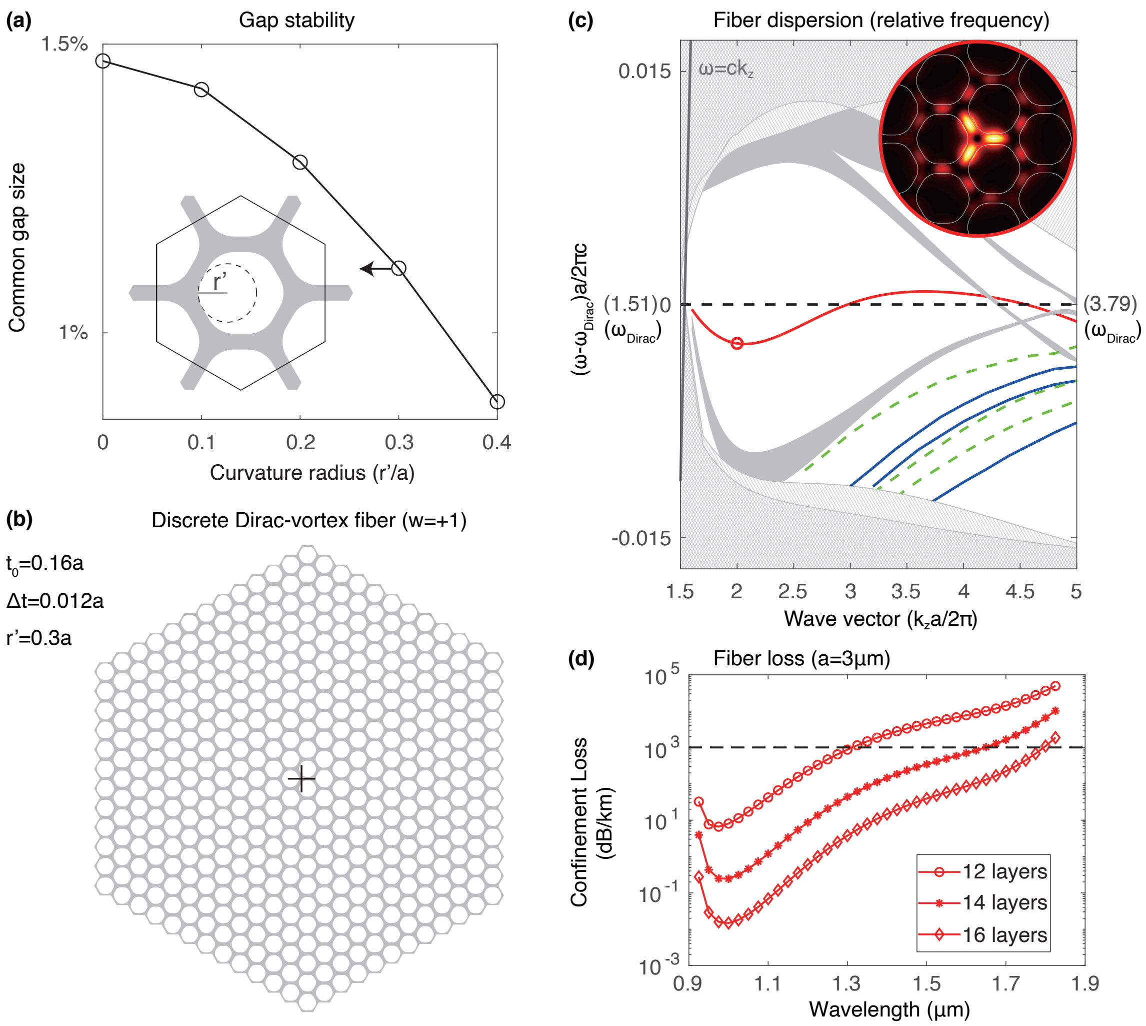

FIG. S4. Design tolerance of structural curvature (r') common in fiber drawing process. (a) Size of common Dirac-vortex bandgap, for all ϕ at $k_z a/2\pi = 2$, as a function of the curvature radius of corners. Inset: supercell with $r' = 0.3a$ and $t_0 = 0.16a$. (b) Fiber structure with $r' = 0.3a$ by adding corner material to the discrete fiber in Fig. 5(b) in the main-text. (c) Band diagram with a frequency reference to the original nodal-line. The dispersion is similar to that in Fig. 5(c). Inset shows the mode profile ($\hat{\boldsymbol{z}} \cdot \mathrm{Re}[\boldsymbol{E}^* \times \boldsymbol{H}]$). (d) Confinement loss of the topological mode. The wavelength range corresponds to the wave vector in (c) for $k_z a/2\pi$ from 1.6 to 4.2.

V. OPERATION ABOVE LIGHT LINE

One key feature of PCFs is the ability to support bandgaps above the light line for hollow-core modes. The topological PCF can operate above the light line as well. In Fig. S5, we pick a particular set of geometric parameters ($t_0 = 0.12a$, $\Delta t = 0.01a$) to push the topological bandgap, and the topological guiding mode, into the light cone. We also calculate the modal concentration factor in air for the topological mode. The maximum air fraction is about 75% for this design, where the effective modal index $n_{\text{eff}} < 1$. In order to have more bandwidth above the light line, we need to improve the frequency isolation of the nodal line degeneracy for low frequencies, so the project bulk bands can remain gapped. This should be possible by increasing the refractive index of glass or explore a different lattice structure.

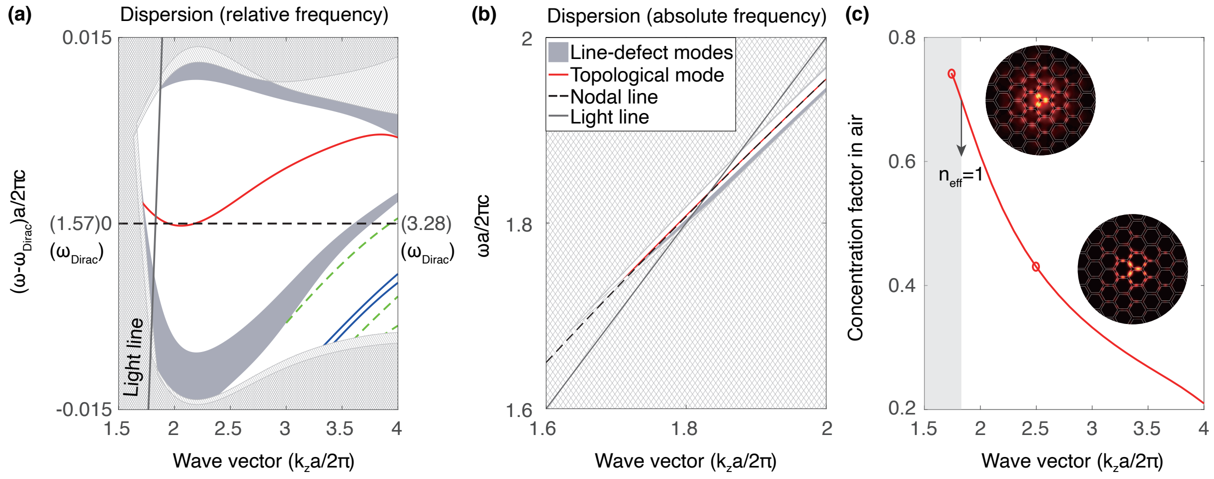

FIG. S5. Topological fiber mode above light line. (a) Band diagram of a discrete Dirac-vortex fiber with $t_0 = 0.12a$ and $\Delta t = 0.01a$ (the discrete version of four tubes). (b) Dispersion of absolute frequency near the light line (c) Concentration of the intensity of the mode in air. Insets: mode intensity at $k_z a/2\pi = 1.74$ and 2.5.

[1] Ramin Beravat, Gordon KL Wong, Michael H Frosz, Xiao Ming Xi, and Philip St J Russell, "Twist-induced guidance in coreless photonic crystal fiber: A helical channel for light," Science Advances **2**, e1601421 (2016).

[2] Niels Asger Mortensen and Martin Dybendal Nielsen, "Modeling of realistic cladding structures for air-core photonic bandgap fibers," Optics letters **29**, 349–351 (2004).

[3] Francesco Poletti, Neil GR Broderick, David James Richardson, and Tanya Mary Monro, "The effect of core asymmetries on the polarization properties of hollow core photonic bandgap fibers," Optics Express **13**, 9115–9124 (2005).
